# Supplementary material for: A broad-spectrum phage-encoded mechanism to disarm bacterial type IV filaments
Source: PLoS Pathog. 2026 Jul 27;22(7):e1014478. doi: 10.1371/journal.ppat.1014478 (PMC13436744; doi:10.1371/journal.ppat.1014478)
Supplement: Supplementary file 1 [file ppat.1014478.s011.pdf]

Roberge et al. Supplementary File S2. Labelled raw images

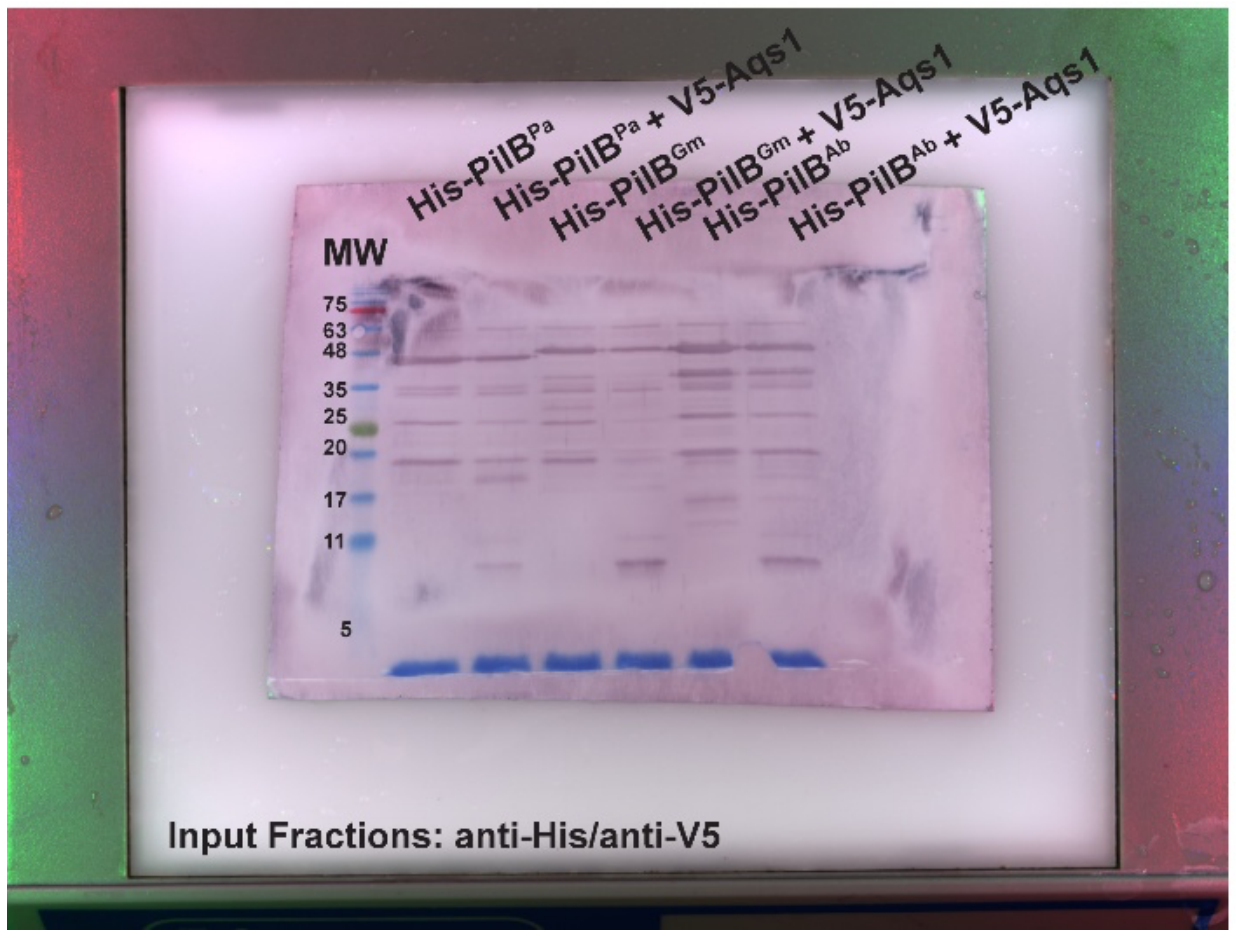

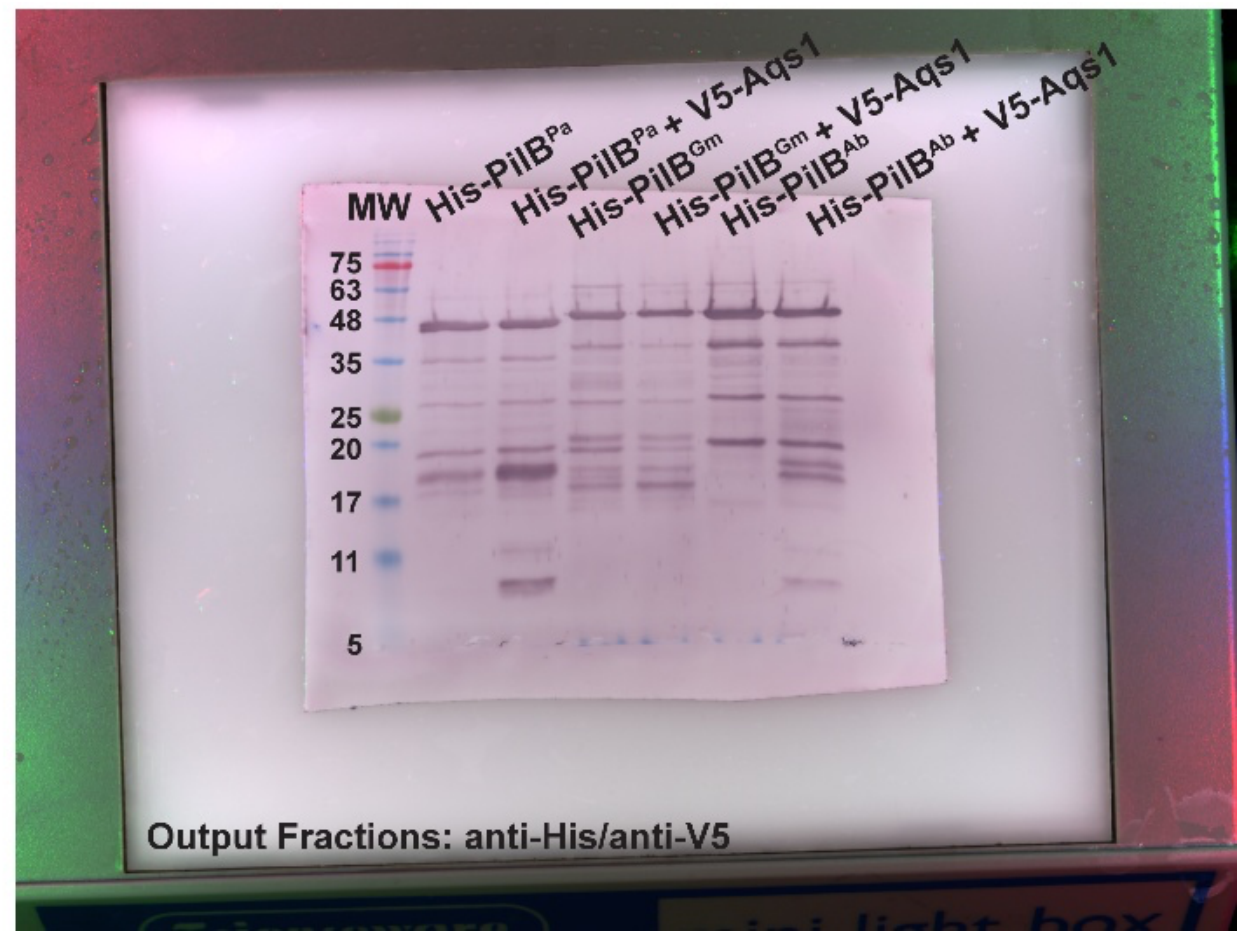

Fig 1D

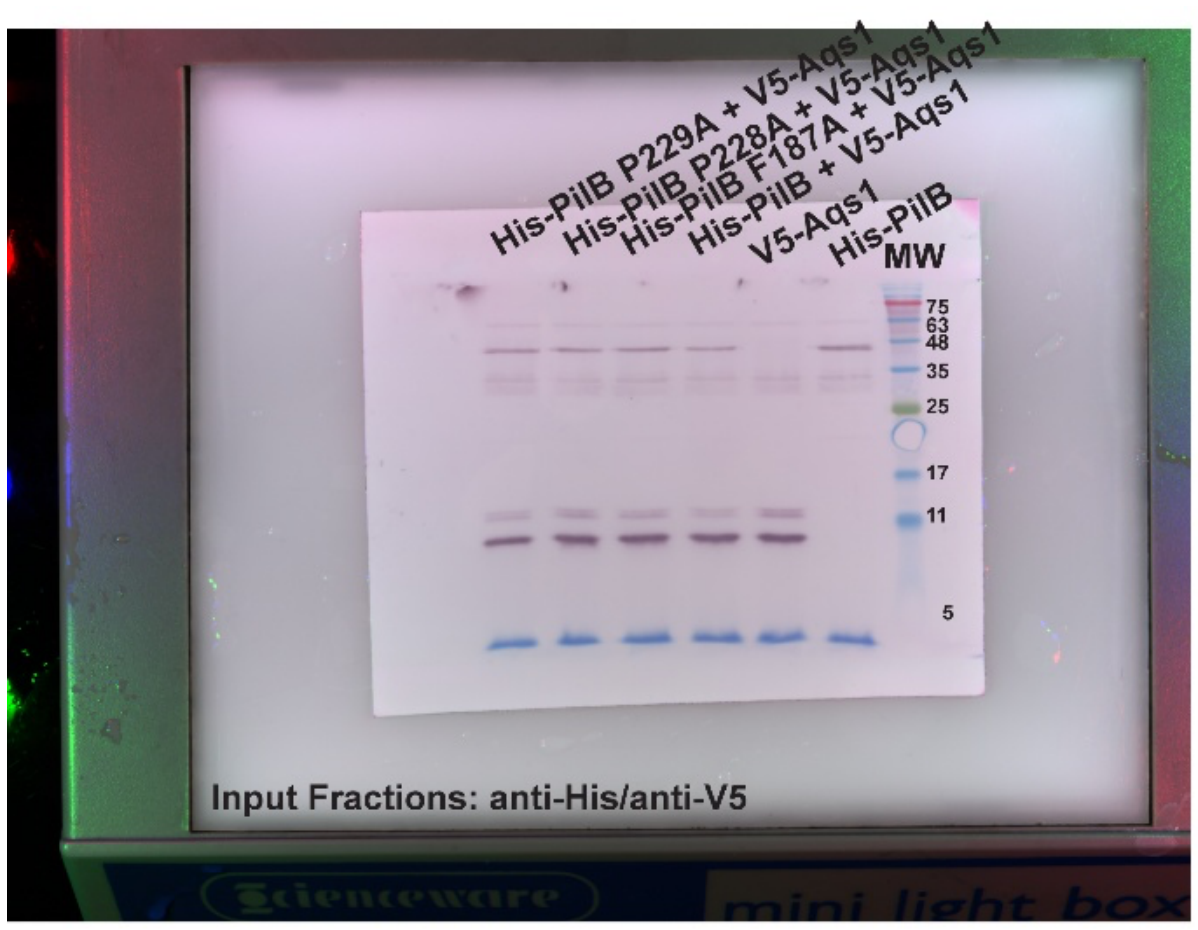

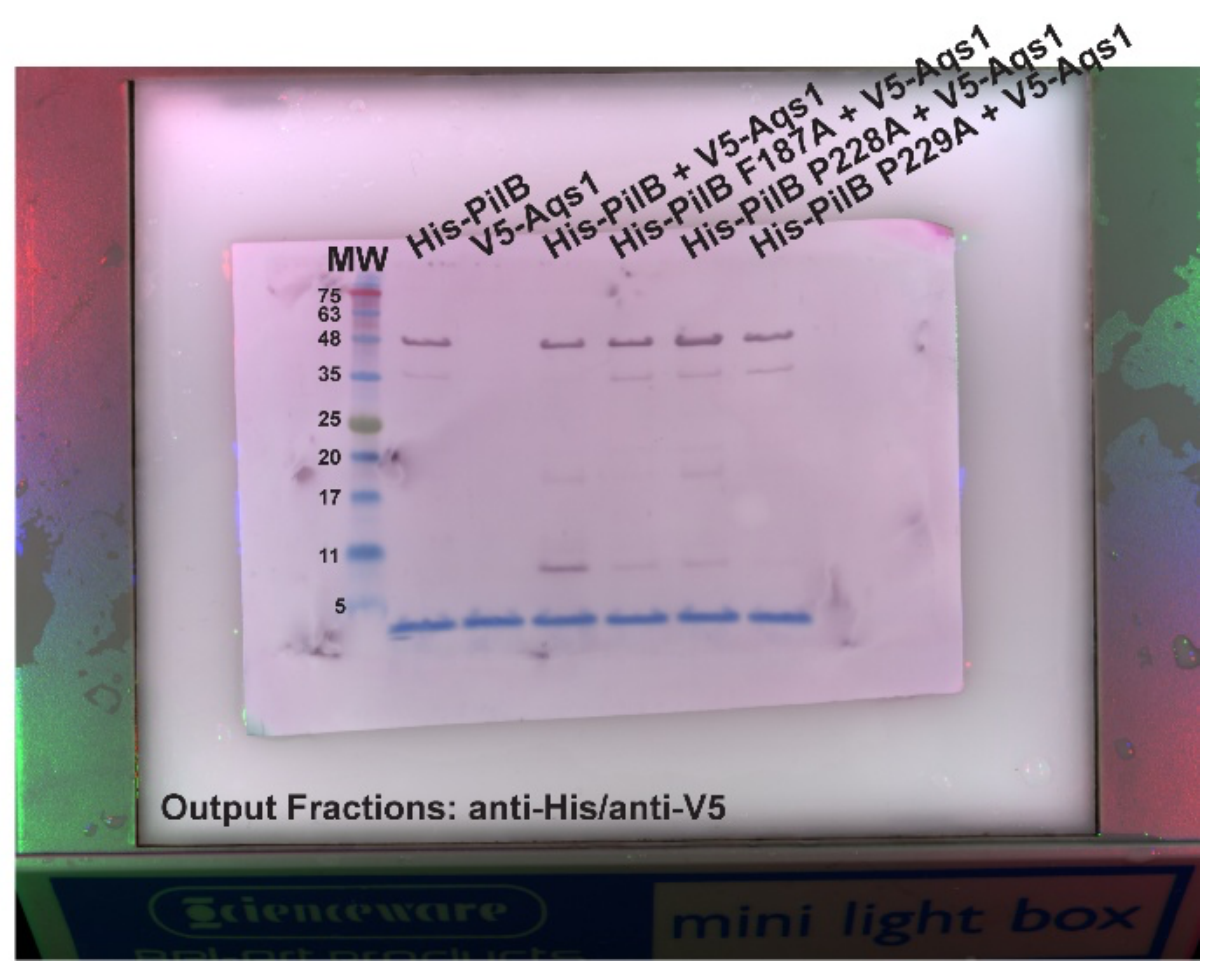

Fig 3C

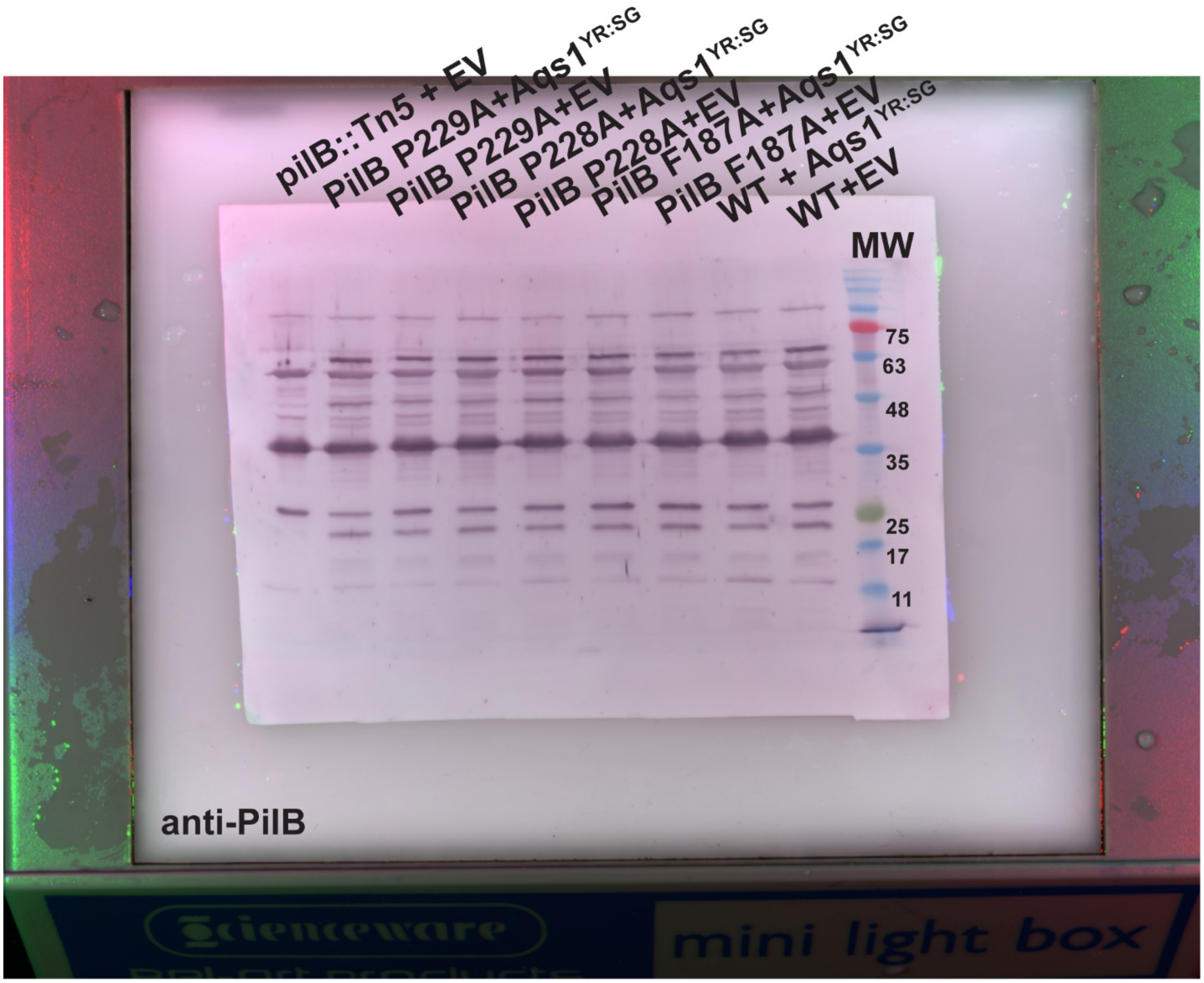
pilB::Tn5 + EV
PilB P229A+Aqs1YR:SG
PilB P229A+EV
PilB P228A+Aqs1YR:SG
PilB P228A+EV
PilB F187A+Aqs1YR:SG
PilB F187A+EV
WT + Aqs1YR:SG
WT+EV
MW
75
63
48
35
25
17
11
anti-PilB
mini light box

Fig 4C

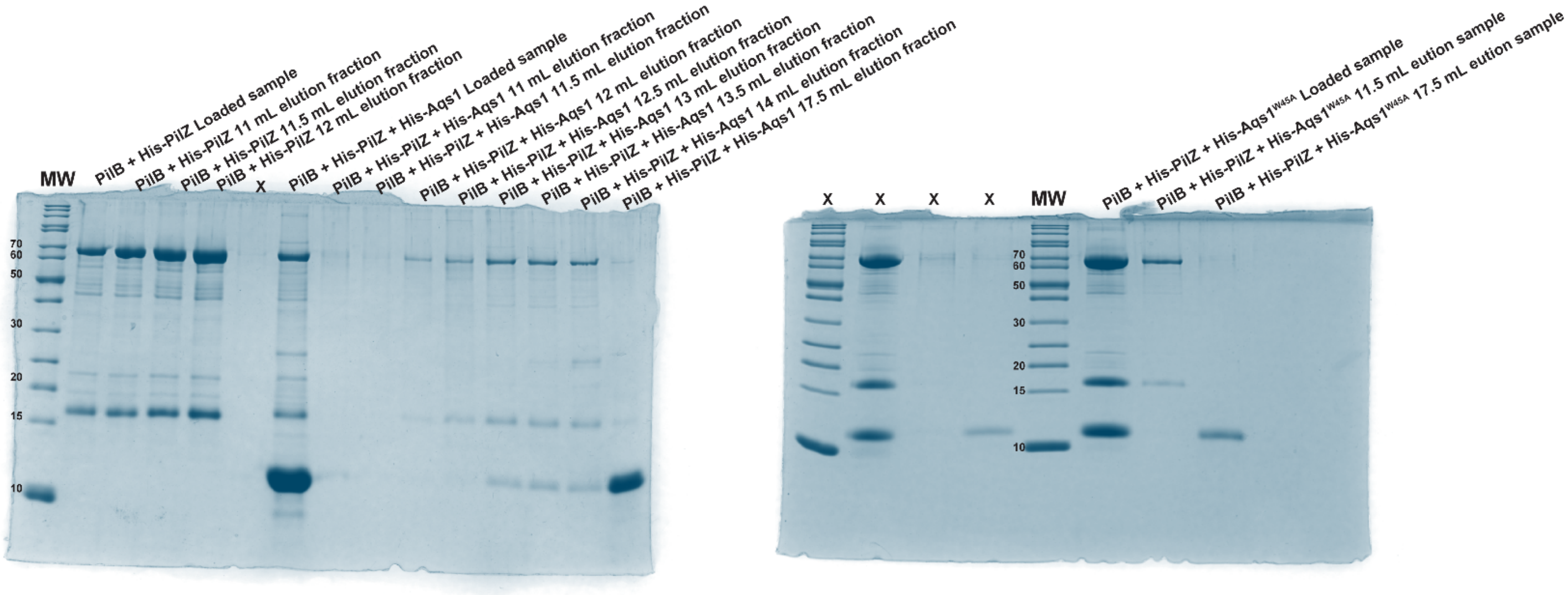
MW
PilB + His-PilZ Loaded sample
PilB + His-PilZ 11 mL elution fraction
PilB + His-PilZ 11.5 mL elution fraction
PilB + His-PilZ 12 mL elution fraction
X
PilB + His-PilZ + His-Aqs1 Loaded sample
PilB + His-PilZ + His-Aqs1 11 mL elution fraction
PilB + His-PilZ + His-Aqs1 11.5 mL elution fraction
PilB + His-PilZ + His-Aqs1 12 mL elution fraction
PilB + His-PilZ + His-Aqs1 12.5 mL elution fraction
PilB + His-PilZ + His-Aqs1 13 mL elution fraction
PilB + His-PilZ + His-Aqs1 13.5 mL elution fraction
PilB + His-PilZ + His-Aqs1 14 mL elution fraction
PilB + His-PilZ + His-Aqs1 17.5 mL elution fraction
70
60
50
30
20
15
10
X
X
X
X
MW
PilB + His-PilZ + His-Aqs1^{W45A} Loaded sample
PilB + His-PilZ + His-Aqs1^{W45A} 11.5 mL eution sample
PilB + His-PilZ + His-Aqs1^{W45A} 17.5 mL eution sample
70
60
50
30
20
15
10

Fig 4E

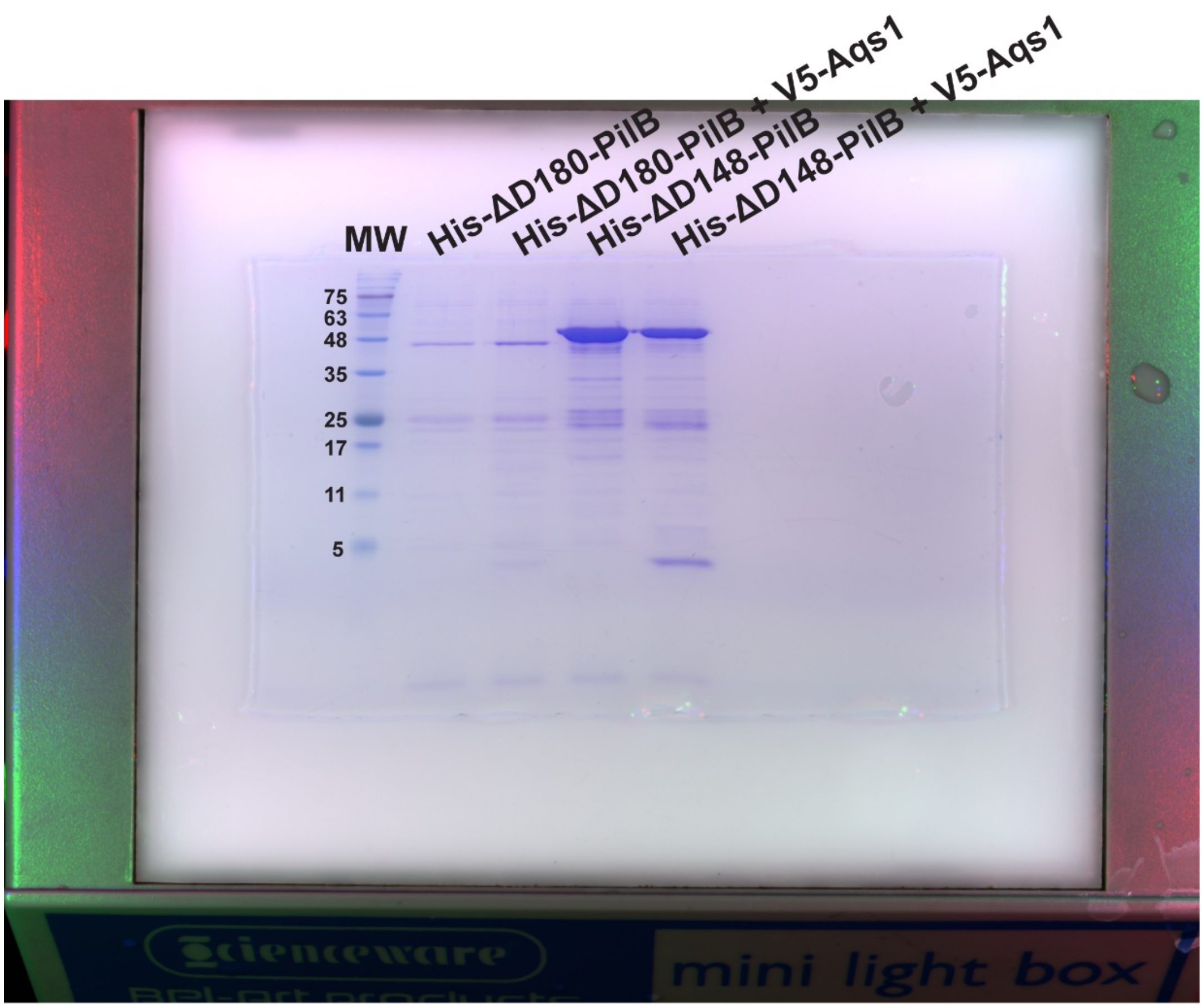
MW
His-ΔD180-PilB
His-ΔD180-PilB + V5-Aqs1
His-ΔD148-PilB
His-ΔD148-PilB + V5-Aqs1
75
63
48
35
25
17
11
5
mini light box

Fig 5B

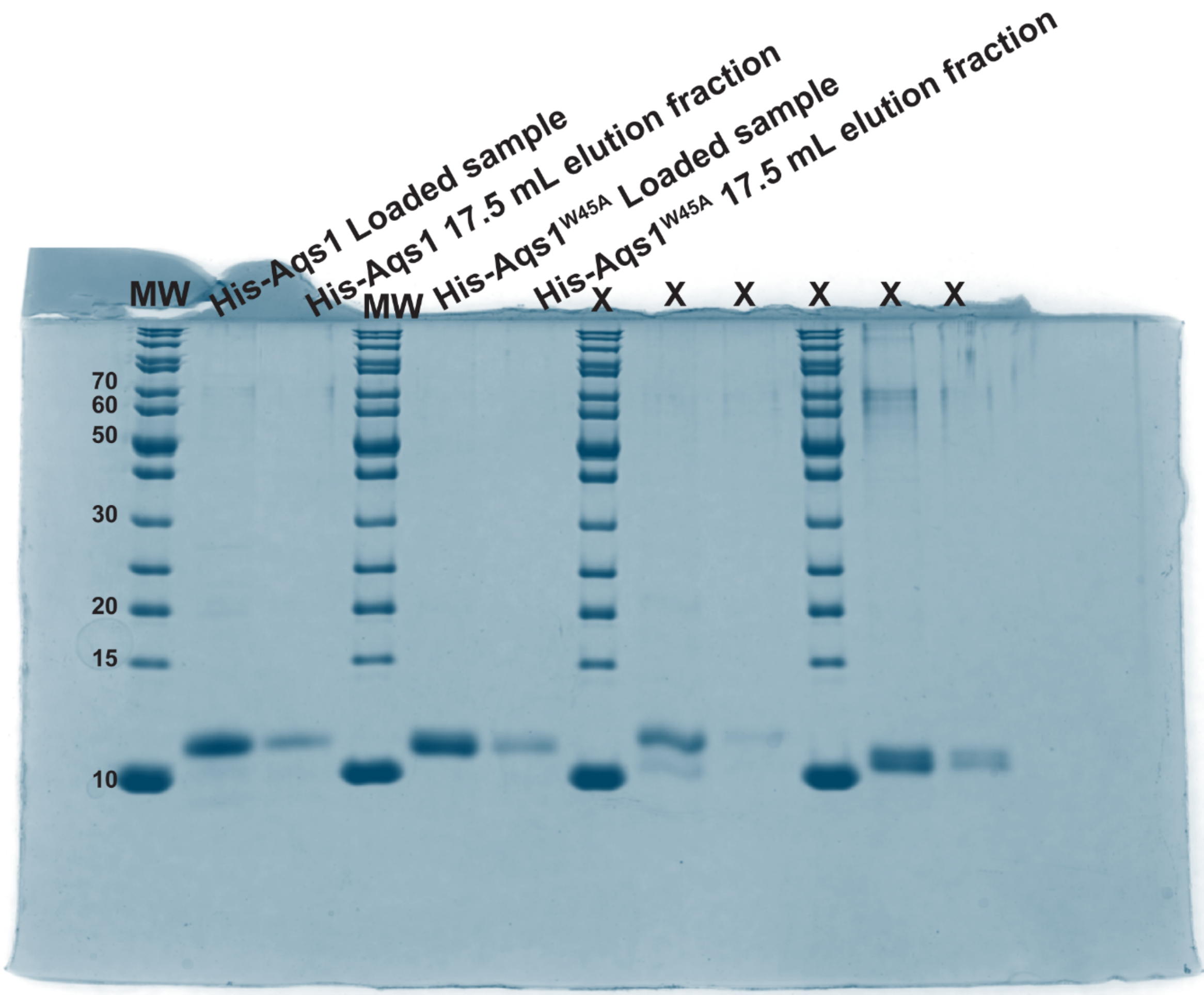
MW
His-Aqs1 Loaded sample
His-Aqs1 17.5 mL elution fraction
MW
His-Aqs1W45A Loaded sample
His-Aqs1W45A 17.5 mL elution fraction
X
X
X
X
X
X
70
60
50
30
20
15
10

Fig S5

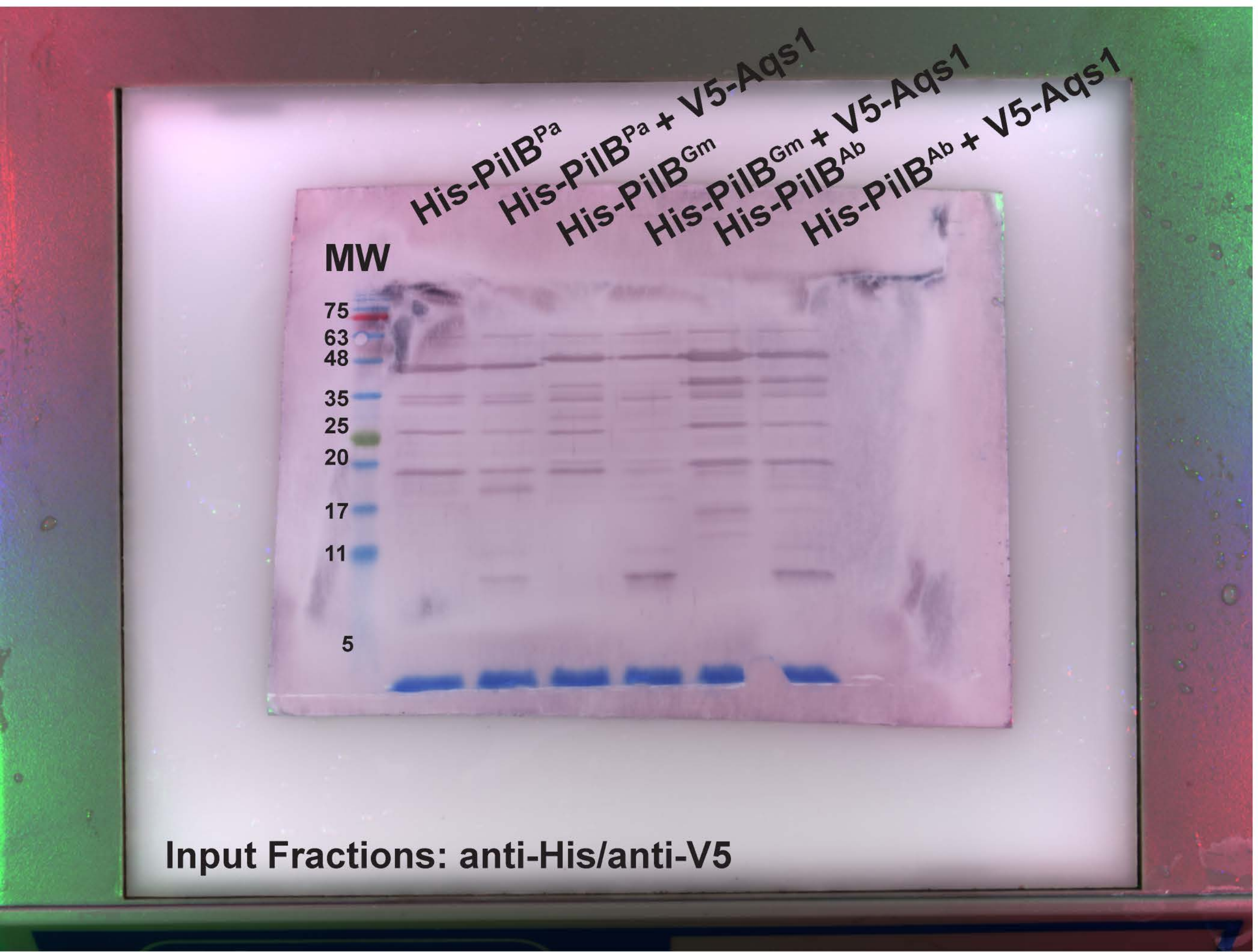
MW
His-PilB^Pa
His-PilB^Pa + V5-Aqs1
His-PilB^Gm
His-PilB^Gm + V5-Aqs1
His-PilB^Ab
His-PilB^Ab + V5-Aqs1
75
63
48
35
25
20
17
11
5
Input Fractions: anti-His/anti-V5

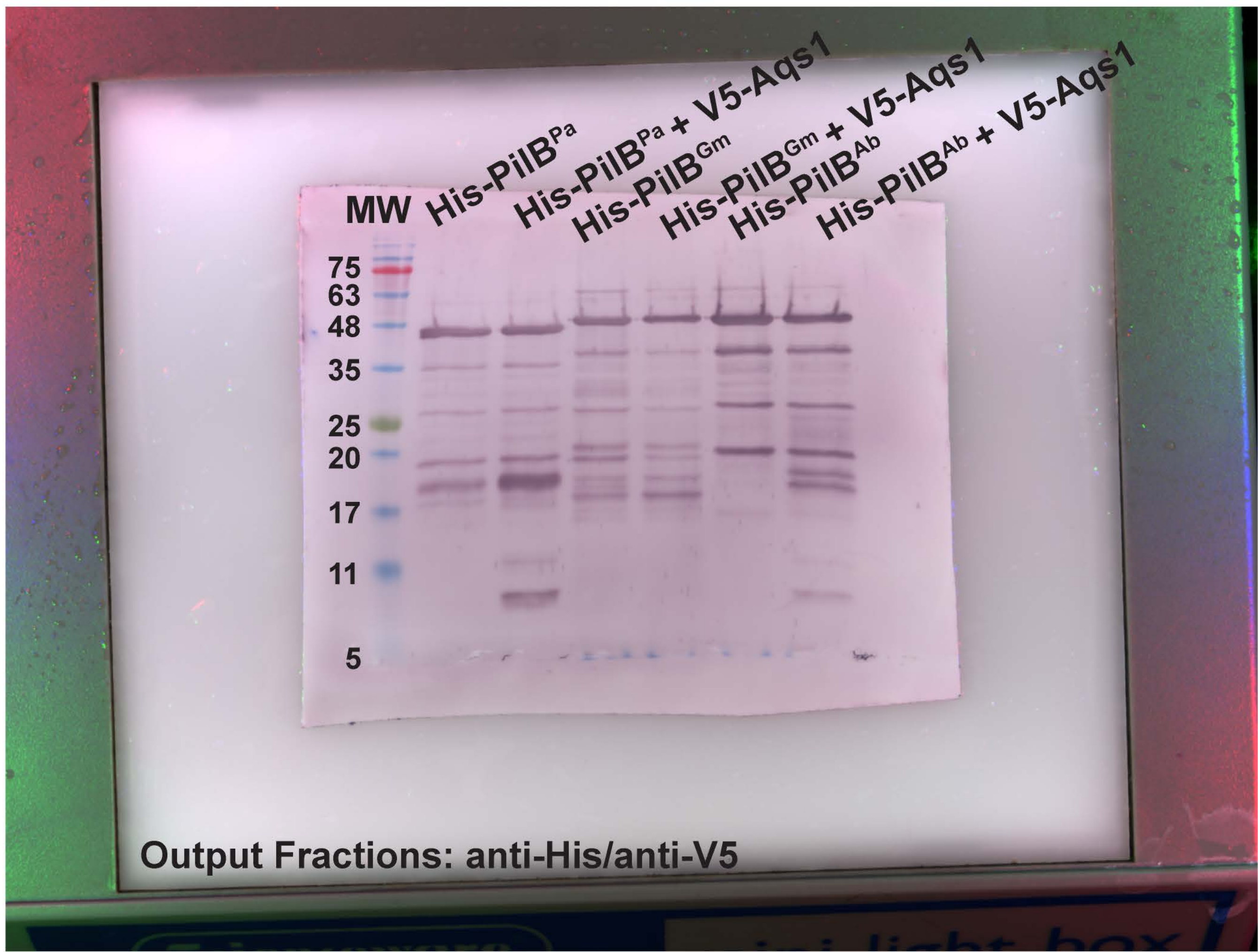
MW
His-PilB^Pa
His-PilB^Pa + V5-Aqs1
His-PilB^Gm
His-PilB^Gm + V5-Aqs1
His-PilB^Ab
His-PilB^Ab + V5-Aqs1
75
63
48
35
25
20
17
11
5
Output Fractions: anti-His/anti-V5

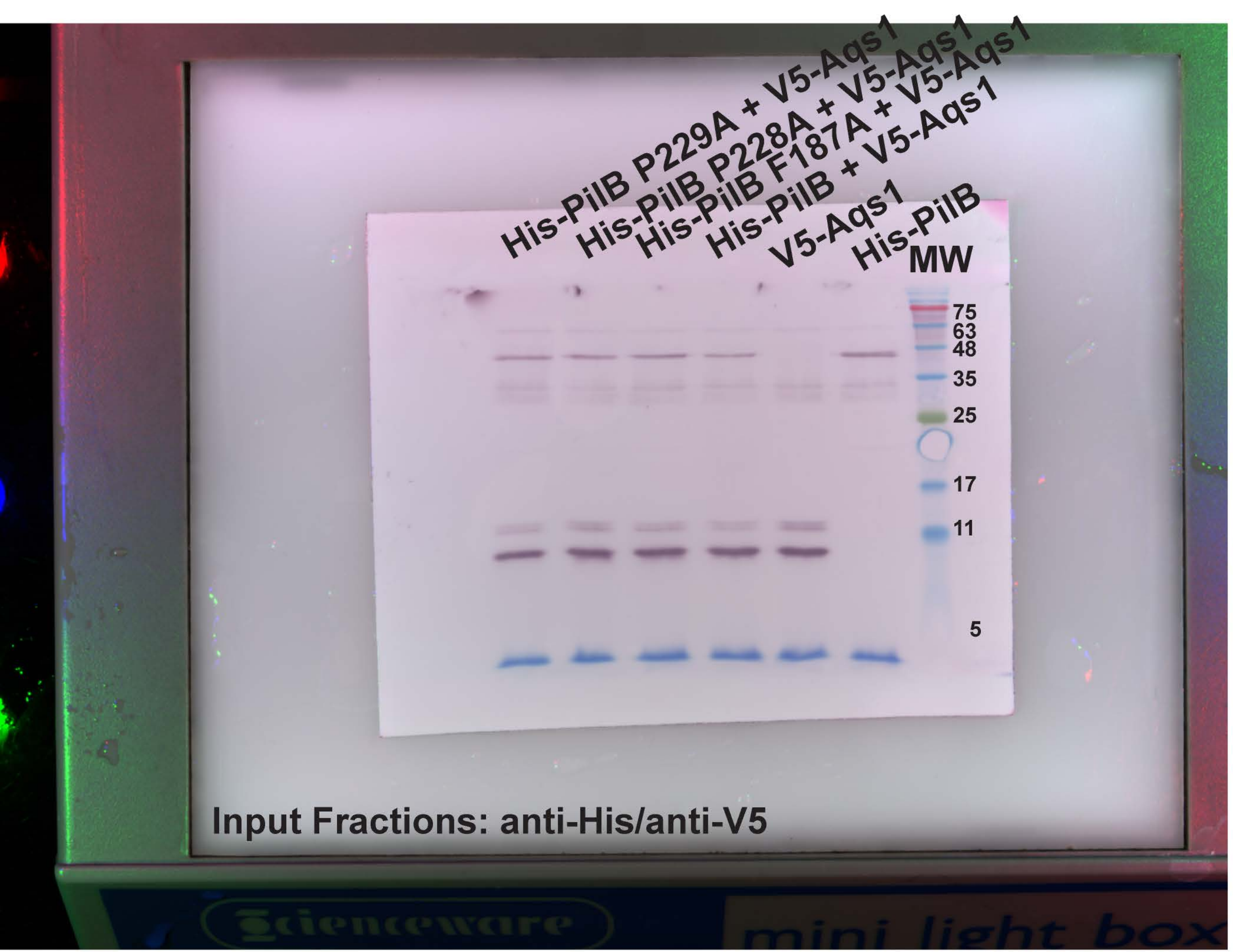

His-PilB P229A + V5-Aqs1
His-PilB P228A + V5-Aqs1
His-PilB F187A + V5-Aqs1
His-PilB + V5-Aqs1
V5-Aqs1
His-PilB
MW
75
63
48
35
25
17
11
5
Input Fractions: anti-His/anti-V5

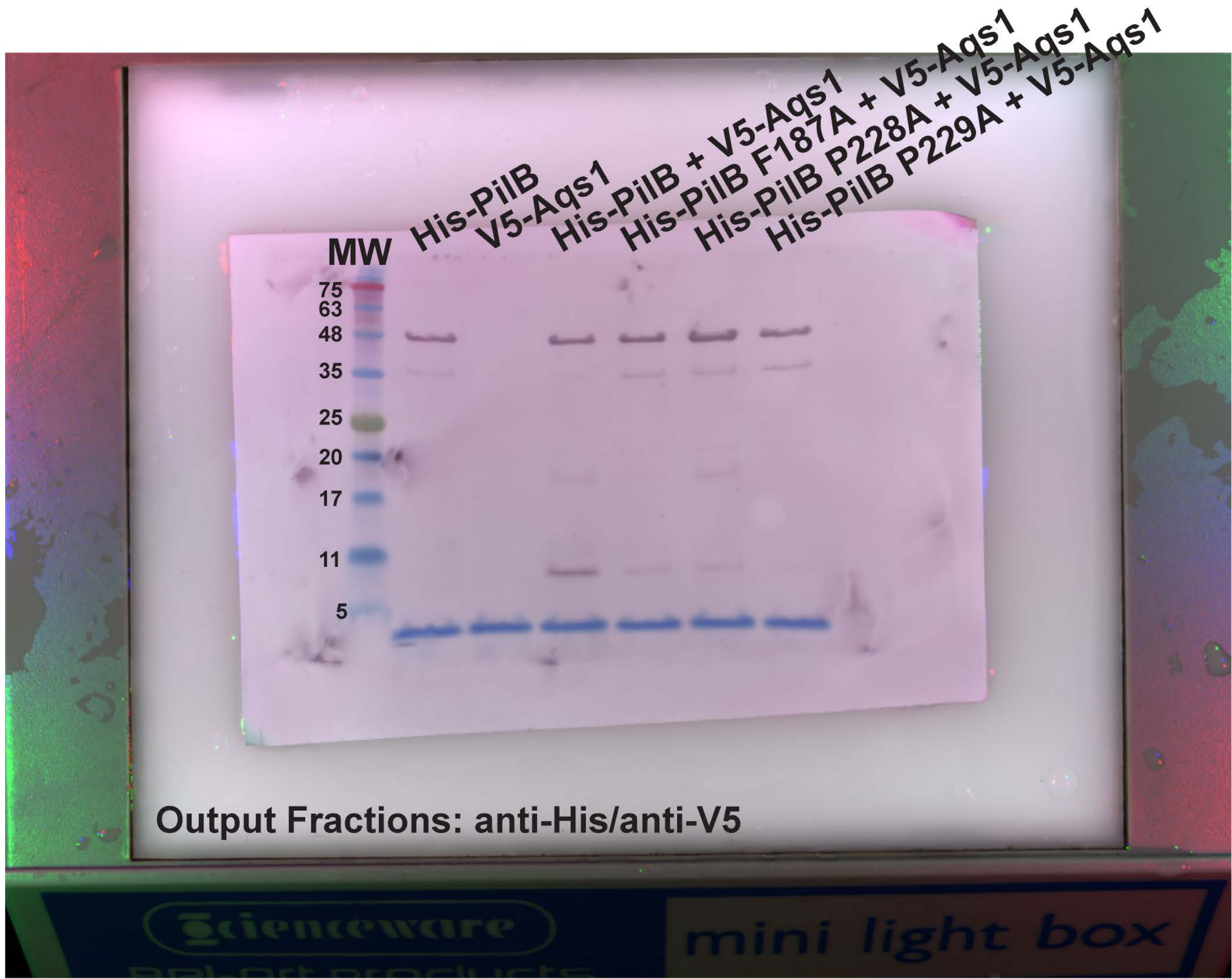

MW
His-PilB
V5-Aqs1
His-PilB + V5-Aqs1
His-PilB F187A + V5-Aqs1
His-PilB P228A + V5-Aqs1
His-PilB P229A + V5-Aqs1
75
63
48
35
25
20
17
11
5
Output Fractions: anti-His/anti-V5

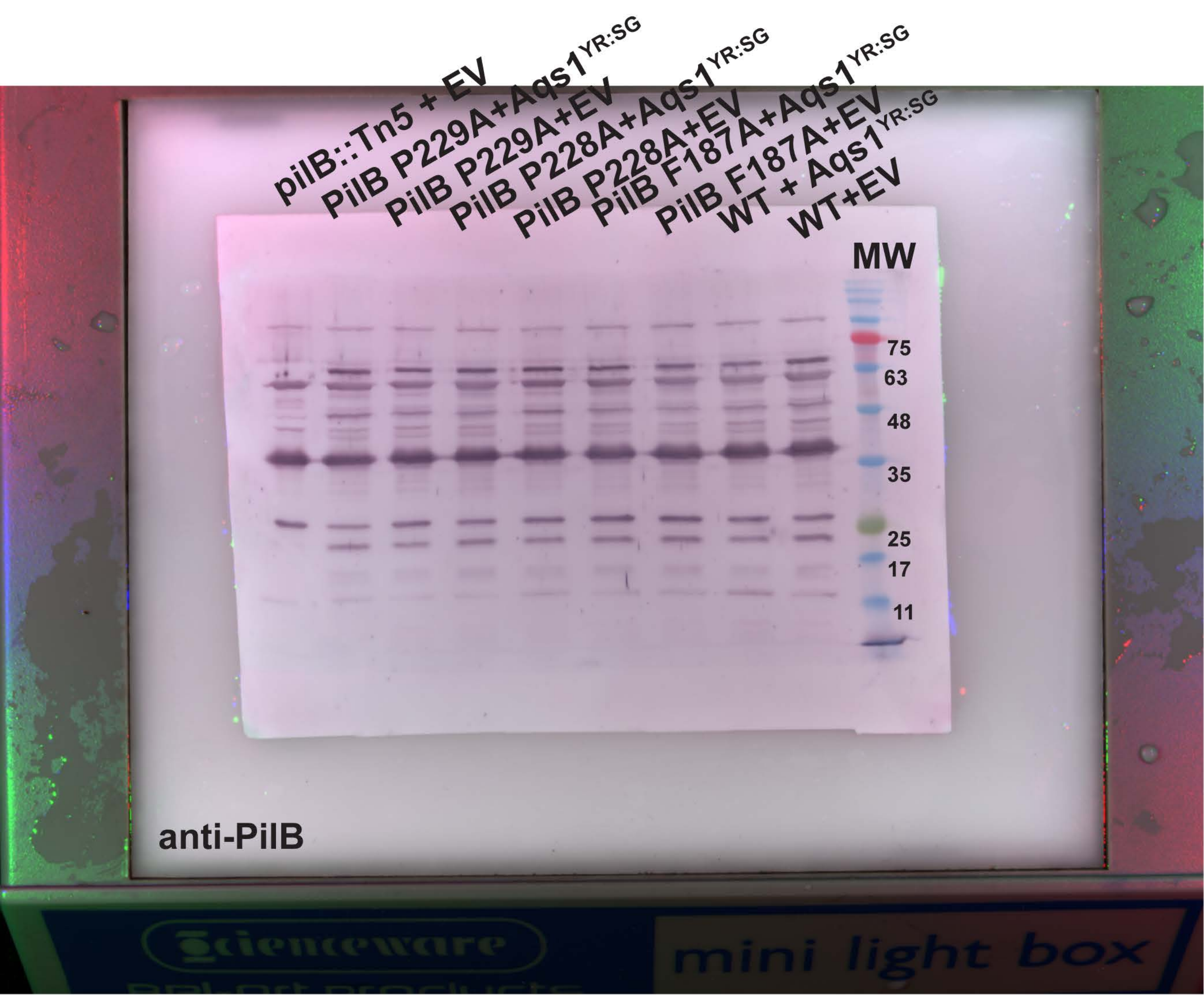

pilB::Tn5 + EV
PilB P229A+Aqs1^YR:SG
PilB P229A+EV
PilB P228A+Aqs1^YR:SG
PilB P228A+EV
PilB F187A+Aqs1^YR:SG
PilB F187A+EV
WT + Aqs1^YR:SG
WT+EV
MW
75
63
48
35
25
17
11
anti-PilB
Scienceware
mini light box

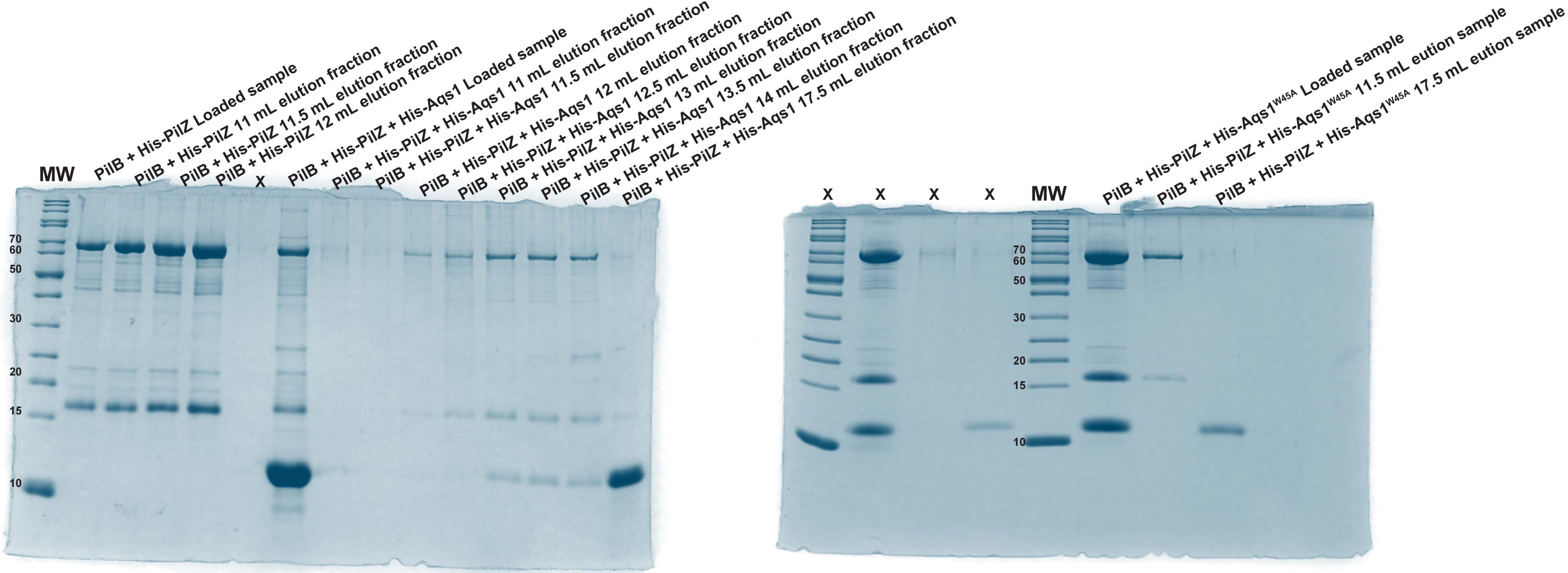
MW
PilB + His-PilZ Loaded sample
PilB + His-PilZ 11 mL elution fraction
PilB + His-PilZ 11.5 mL elution fraction
PilB + His-PilZ 12 mL elution fraction
X
PilB + His-PilZ + His-Aqs1 Loaded sample
PilB + His-PilZ + His-Aqs1 11 mL elution fraction
PilB + His-PilZ + His-Aqs1 11.5 mL elution fraction
PilB + His-PilZ + His-Aqs1 12 mL elution fraction
PilB + His-PilZ + His-Aqs1 12.5 mL elution fraction
PilB + His-PilZ + His-Aqs1 13 mL elution fraction
PilB + His-PilZ + His-Aqs1 13.5 mL elution fraction
PilB + His-PilZ + His-Aqs1 14 mL elution fraction
PilB + His-PilZ + His-Aqs1 17.5 mL elution fraction
70
60
50
30
20
15
10
X
X
X
X
MW
PilB + His-PilZ + His-Aqs1^{W45A} Loaded sample
PilB + His-PilZ + His-Aqs1^{W45A} 11.5 mL eution sample
PilB + His-PilZ + His-Aqs1^{W45A} 17.5 mL eution sample
70
60
50
30
20
15
10

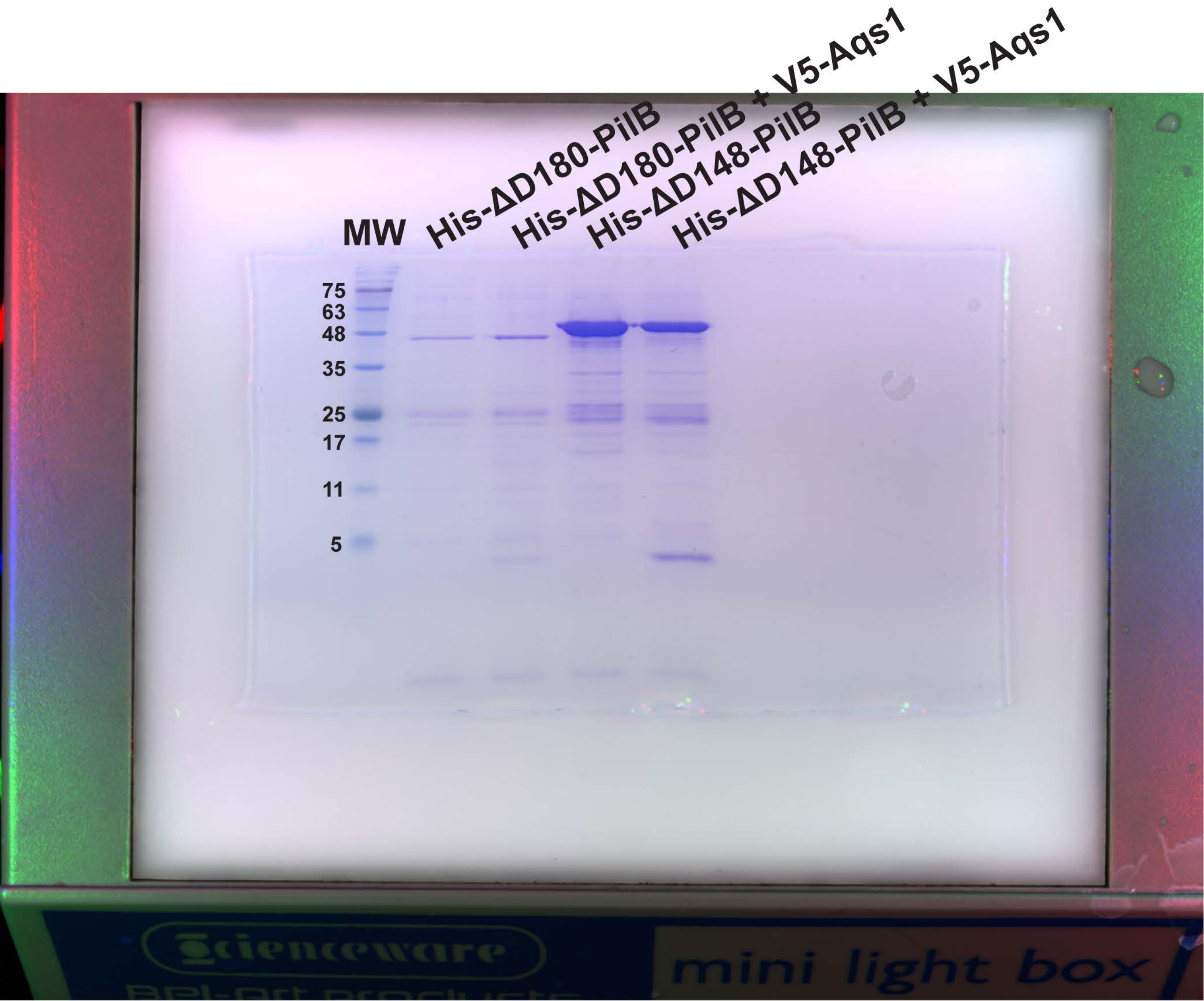
MW
His-ΔD180-PilB
His-ΔD180-PilB + V5-Aqs1
His-ΔD148-PilB
His-ΔD148-PilB + V5-Aqs1
75
63
48
35
25
17
11
5
Scienceware
mini light box

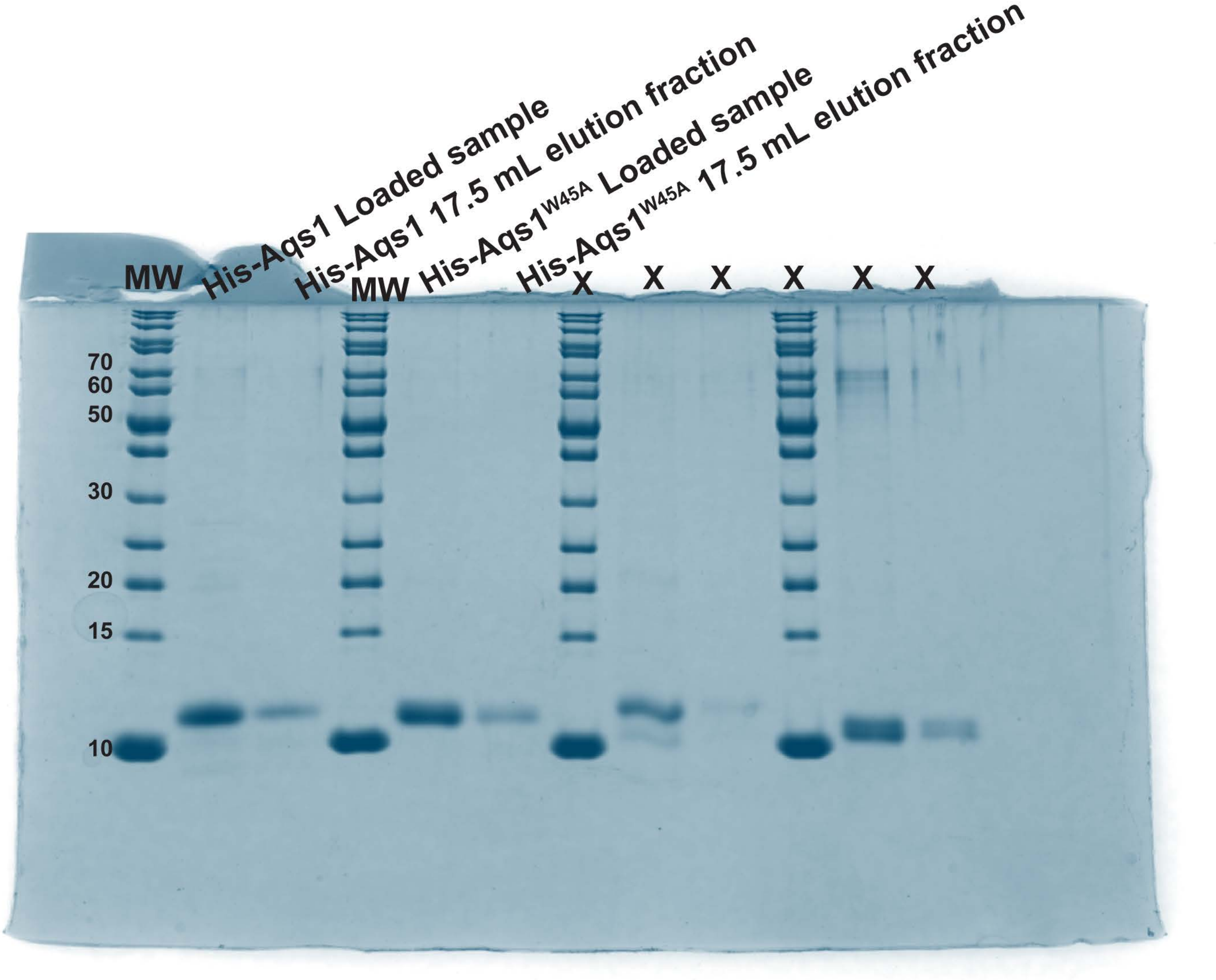
MW
His-Aqs1 Loaded sample
His-Aqs1 17.5 mL elution fraction
MW
His-Aqs1W45A Loaded sample
His-Aqs1W45A 17.5 mL elution fraction
X
X
X
X
X
X
70
60
50
30
20
15
10
