# Supplementary material for: A broad-spectrum phage-encoded mechanism to disarm bacterial type IV filaments
Source: PLoS Pathog. 2026 Jul 27;22(7):e1014478. doi: 10.1371/journal.ppat.1014478 (PMC13436744; doi:10.1371/journal.ppat.1014478)
Supplement: Supplementary file 2 [file ppat.1014478.s001.docx]

**S1 Table: All strains and plasmids used in this study.**

| **Strains** | | |
| --- | --- | --- |
| **Strain name** | **Genotype** | **Source** |
| ***E. coli* strains** | | |
| DH5α | *F-φ80lacZΔM15* Δ(*lacZYA-argF*)*U169 recA1 endA1 hsdR17*(*rk−, mk+*) *phoA supE44 thi-1 gyrA96 relA1 λ−* | Invitrogen |
| BL21 (DE3) | *fhuA2 [lon] ompT gal [dcm] ΔhsdS* | New England Biolabs |
| BL21 pLysS | *F–, ompT, hsdS_B_ (r_B_–, m_B_–), dcm, gal, λ(DE3), pLysS, Cm^r^* | Novagen |
| BTH101 | Bacterial 2-hybrid strain | Euromedex |
| SM10 | *thi-1 thr leu tonA lacY supE recA*::*RP4-2-Tc::Mu* (*KmR*) | Invitrogen |
| PilS/PilS | BTH101 with PilS in pUT18C and pKT25 vectors | ^1^ |
| T18/T25 | BTH101 with empty pUT18C and pKT25 vectors | This study |
| PilB/PilB | BTH101 with PilB in pUT18C and pKT25 vectors | This study |
| PilB-T18/Aqs1-T25 | BTH101 with PilB in pUT18C and Aqs1 in pKT25 vectors | This study |
| PilB-T25/Aqs1-T18 | BTH101 with PilB in pKT25 and Aqs1 in pUT18C vectors | This study |
| PilB-T18/Aqs1 YR:SG -T25 | BTH101 with PilB in pUT18C and Aqs1 YR:SG in pKT25 vectors | This study |
| PilB-T25/Aqs1 YR:SG -T18 | BTH101 with PilB in pKT25 and Aqs1 YR:SG in pUT18C vectors | This study |
| LasR-T18/Aqs1-T25 | BTH101 with LasR in pUT18C and Aqs1 in pKT25 vectors | This study |
| LasR-T25/Aqs1-T18 | BTH101 with LasR in pKT25 and Aqs1 in pUT18C vectors | This study |
| LasR-T18/Aqs1 YR:SG -T25 | BTH101 with LasR in pUT18C and Aqs1 YR:SG in pKT25 vectors | This study |
| LasR-T25/Aqs1 YR:SG -T18 | BTH101 with LasR in pKT25 and Aqs1 YR:SG in pUT18C vectors | This study |
| PilB P183A-T18/Aqs1-T25 | BTH101 with PilB P183A in pUT18C and Aqs1 in pKT25 vectors | This study |
| PilB P183A-T25/Aqs1-T18 | BTH101 with PilB P183A in pKT25 and Aqs1 in pUT18C vectors | This study |
| PilB F187A-T18/Aqs1-T25 | BTH101 with PilB F187A in pUT18C and Aqs1 in pKT25 vectors | This study |
| PilB F187A-T25/Aqs1-T18 | BTH101 with PilB F187A in pKT25 and Aqs1 in pUT18C vectors | This study |
| PilB P228A-T18/Aqs1-T25 | BTH101 with PilB P228A in pUT18C and Aqs1 in pKT25 vectors | This study |
| PilB P228A-T25/Aqs1-T18 | BTH101 with PilB P228A in pKT25 and Aqs1 in pUT18C vectors | This study |
| PilB P229A-T18/Aqs1-T25 | BTH101 with PilB P229A in pUT18C and Aqs1 in pKT25 vectors | This study |
| PilB P229A-T25/Aqs1-T18 | BTH101 with PilB P229A in pKT25 and Aqs1 in pUT18C vectors | This study |
| PilB L232A-T18/Aqs1-T25 | BTH101 with PilB L232A in pUT18C and Aqs1 in pKT25 vectors | This study |
| PilB L232A-T25/Aqs1-T18 | BTH101 with PilB L232A in pKT25 and Aqs1 in pUT18C vectors | This study |
| PilB I236A-T18/Aqs1-T25 | BTH101 with PilB I236A in pUT18C and Aqs1 in pKT25 vectors | This study |
| PilB I236A-T25/Aqs1-T18 | BTH101 with PilB I236A in pKT25 and Aqs1 in pUT18C vectors | This study |
| pETDuet-1 ΔN1D (ΔD180) PilB | BL21 pLysS with N-terminally truncated His_6_-tagged PilB in the first site and empty second site for heterologous expression in *E. coli* | This study |
| pETDuet-1 Aqs1 | BL21 pLysS with an empty first site and V5-tagged Aqs1 in the second site for heterologous expression in *E. coli* | This study |
| pETDuet-1 ΔN1D PilB/Aqs1 | BL21 pLysS with N-terminally truncated His_6_-tagged PilB in the first site and V5-tagged Aqs1 in the second site for heterologous expression in *E. coli* | This study |
| pETDuet-1 ΔN1D PilB F187A/Aqs1 | BL21 pLysS with N-terminally truncated His_6_-tagged PilB F187A in the first site and V5-tagged Aqs1 in the second site for heterologous expression in *E. coli* | This study |
| pETDuet-1 ΔN1D PilB P228A/Aqs1 | BL21 pLysS with N-terminally truncated His_6_-tagged PilB P228A in the first site and V5-tagged Aqs1 in the second site for heterologous expression in *E. coli* | This study |
| pETDuet-1 ΔN1D PilB P229A/Aqs1 | BL21 pLysS with N-terminally truncated His_6_-tagged PilB P229A in the first site and V5-tagged Aqs1 in the second site for heterologous expression in *E. coli* | This study |
| PilB-T18/Tip-T25 | BTH101 with PilB in pUT18C and Tip in pKT25 vectors | This study |
| PilB-T25/Tip-T18 | BTH101 with PilB in pKT25 and Tip in pUT18C vectors | This study |
| PilB pCDF PilZ pETDuet-1 | BL21 with PilB in pCDF and His_6_-PilZ in pETDuet-1 first site with empty second site for heterologous expression in *E. coli* | This study |
| Aqs1 p15TVL | BL21 with His_6_-Aqs1 in p15TVL for heterologous expression in *E. coli* | ^2^ |
| Aqs1 W45A p15TVL | BL21 with His_6_-Aqs1 W45A in p15TVL for heterologous expression in *E. coli* | ^2^ |
| PilB+PilZ+Aqs1 | BL21 with His_6_-Aqs1 and PilB in pETDuet-1 first and second sites respectively and PilZ in pACYDuet second site with empty first site | This study |
| pETDuet-1 ΔT148 PilB | BL21 pLysS with linker intact N-terminally truncated His_6_-tagged PilB in the first site and empty second site for heterologous expression in *E. coli* | This study |
| pETDuet-1 ΔT148 PilB/Aqs1 | BL21 pLysS with linker intact N-terminally truncated His_6_-tagged PilB in the first site and V5-tagged Aqs1 in the second site for heterologous expression in *E. coli* | This study |
| PilB ΔT148/PilB ΔT148 | BTH101 with PilB linker intact ΔT148 in pUT18C and pKT25 vectors | This study |
| PilB ΔT148 N mut/ PilB ΔT148 N mut | BTH101 with PilB linker intact ΔT148 in pUT18C and pKT25 vectors | This study |
| PilB N mut /PilB N mut | BTH101 with PilB L154N, L159N, L162N, and I164N in pUT18C and pKT25 vectors | This study |
| PilB ΔD180 N mut -T18/Aqs1-T25 | BTH101 with N-terminally truncated PilB in pUT18C and Aqs1 in pKT25 vectors | This study |
| PilB ΔD180 N mut-T25/Aqs1-T18 | BTH101 with N-terminally truncated PilB in pKT25 and Aqs1 in pUT18C vectors | This study |
| PilB^Gm^/PilB^Gm^ | BTH101 with PilB from *Geobacter metallireducens* in pUT18C and pKT25 vectors | This study |
| PilB^Gm^-T18/Aqs1-T25 | BTH101 with PilB from *Geobacter metallireducens* in pUT18C and Aqs1 pKT25 vectors | This study |
| PilB^Gm^-T25/Aqs1-T18 | BTH101 with PilB from *Geobacter metallireducens* in pKT25 and Aqs1 pUT18C vectors | This study |
| PilB^Ab^/PilB^Ab^ | BTH101 with PilB from *Acinetobacter baylyi* in pUT18C and pKT25 vectors | This study |
| PilB^Ab^-T18/Aqs1-T25 | BTH101 with PilB from *Acinetobacter baylyi* in pUT18C and Aqs1 pKT25 vectors | This study |
| PilB^Ab^-T25/Aqs1-T18 | BTH101 with PilB from *Acinetobacter baylyi* in pKT25 and Aqs1 pUT18C vectors | This study |
| TfpB/TfpB | BTH101 with TfpB from *Acinetobacter baylyi* in pUT18C and pKT25 vectors | This study |
| TfpB-T18/Aqs1-T25 | BTH101 with TfpB from *Acinetobacter baylyi* in pUT18C and Aqs1 pKT25 vectors | This study |
| TfpB-T25/Aqs1-T18 | BTH101 with TfpB from *Acinetobacter baylyi* in pKT25 and Aqs1 pUT18C vectors | This study |
| MshE/MshE | BTH101 with MshE from *Vibrio cholerae* in pUT18C and pKT25 vectors | This study |
| MshE-T18/Aqs1-T25 | BTH101 with MshE from *Vibrio cholerae* in pUT18C and Aqs1 pKT25 vectors | This study |
| MshE-T25/Aqs1-T18 | BTH101 with MshE from *Vibrio cholerae* in pKT25 and Aqs1 pUT18C vectors | This study |
| CpaF/ CpaF | BTH101 with CpaF from *Caulobacter crescentus* in pUT18 and pKNT25 vectors | This study |
| CpaF -T18/Aqs1-T25 | BTH101 with CpaF from *Caulobacter crescentus* in pUT18 and Aqs1 pKT25 vectors | This study |
| CpaF -T25/Aqs1-T18 | BTH101 with CpaF from *Caulobacter crescentus* in pKNT25 and Aqs1 pUT18C vectors | This study |
| pETDuet-1 ΔN1D PilB^Gm^ | BL21 pLysS with linker intact N-terminally truncated His_6_-tagged PilB^Gm^ in the first site and empty second site for heterologous expression in *E. coli* | This study |
| pETDuet-1 ΔN1D PilB^Gm^/Aqs1 | BL21 pLysS with linker intact N-terminally truncated His_6_-tagged PilB^Gm^ in the first site and V5-tagged Aqs1 in the second site for heterologous expression in *E. coli* | This study |
| pETDuet-1 ΔN1D PilB^Ab^ | BL21 pLysS with linker intact N-terminally truncated His_6_-tagged PilB^Ab^ in the first site and empty second site for heterologous expression in *E. coli* | This study |
| pETDuet-1 ΔN1D PilB^Ab^/Aqs1 | BL21 pLysS with linker intact N-terminally truncated His_6_-tagged PilB^Ab^ in the first site and V5-tagged Aqs1 in the second site for heterologous expression in *E. coli* | This study |
| ***P. aeruginosa* strains** | | |
| mPAO1 | WT | ^3^ |
| *pilA*::Tn5 | mPAO1 *pilA* T5 transposon mutant | ^3^ |
| *pilB*::Tn5 | mPAO1 *pilB* T5 transposon mutant | ^3^ |
| Δ*fimX* | mPAO1 Δ*fimX* | ^4^ |
| Δ*fimX* + EV | mPAO1 Δ*fimX* + empty pHERD30T | This study |
| Δ*fimX* + Aqs1 YR:SG | mPAO1 Δ *fimX* + His_6_-Aqs1 YR:SG pHERD30T | This study |
| Δ*pilZ* | mPAO1 Δ*pilZ* | ^4^ |
| mPAO1 + EV | WT *P. aeruginosa* with empty pHERD30T | ^4^ |
| mPAO1 + Aqs1 | WT *P. aeruginosa* with His_6_-Aqs1 in pHERD30T | This study |
| mPAO1 + Aqs1 YR:SG | WT *P. aeruginosa* with His_6_-Aqs1 YR:SG in pHERD30T | This study |
| mPAO1 + Aqs1 F19A | WT *P. aeruginosa* with His_6_-Aqs1 F19A in pHERD30T | This study |
| mPAO1 + Aqs1 W45A | WT *P. aeruginosa* with His_6_-Aqs1 W45A in pHERD30T | This study |
| *pilB*::Tn5 + EV | mPAO1 *pilB* T5 transposon mutant with empty pHERD30T | ^4^ |
| Δ*lasR* + EV | mPAO1 Δ*lasR* with empty pHERD30T | This study |
| *pilB*::Tn5 + PilB | mPAO1 *pilB* T5 transposon mutant with PilB in pHERD30T | ^4^ |
| *pilB*::Tn5 + PilB P183A | mPAO1 *pilB* T5 transposon mutant with PilB P183A in pHERD30T | This study |
| *pilB*::Tn5 + PilB F187A | mPAO1 *pilB* T5 transposon mutant with PilB F187A in pHERD30T | This study |
| *pilB*::Tn5 + PilB P228A | mPAO1 *pilB* T5 transposon mutant with PilB P228A in pHERD30T | This study |
| *pilB*::Tn5 + PilB P229A | mPAO1 *pilB* T5 transposon mutant with PilB P229A in pHERD30T | This study |
| *pilB*::Tn5 + PilB L232A | mPAO1 *pilB* T5 transposon mutant with PilB L232A in pHERD30T | This study |
| *pilB*::Tn5 + PilB I236A | mPAO1 *pilB* T5 transposon mutant with PilB I236A in pHERD30T | This study |
| PilB F187A + EV | mPAO1 with chromosomal F187A mutation with empty pHERD30T | This study |
| PilB P228A + EV | mPAO1 with chromosomal P228A mutation with empty pHERD30T | This study |
| PilB P229A + EV | mPAO1 with chromosomal P229A mutation with empty pHERD30T | This study |
| PilB F187A + Aqs1 YR:SG | mPAO1 with chromosomal F187A mutation with His_6_-Aqs1 YR:SG in pHERD30T | This study |
| PilB P228A + Aqs1 YR:SG | mPAO1 with chromosomal P228A mutation with His_6_-Aqs1 YR:SG in pHERD30T | This study |
| PilB P229A + Aqs1 YR:SG | mPAO1 with chromosomal P229A mutation with His_6_-Aqs1 YR:SG in pHERD30T | This study |
| PilA A86C + EV | mPAO1 PilA A86C with empty pHERD30T | This study |
| PilA A86C + Aqs1 YR:SG | mPAO1 PilA A86C with His_6_-Aqs1 YR:SG in pHERD30T | This study |
| PilB F187A PilA A86C + EV | mPAO1 PilB F187A PilA A86C with empty pHERD30T | This study |
| PilB F187A PilA A86C + Aqs1 YR:SG | mPAO1 PilB F187A PilA A86C with His_6_-Aqs1 YR:SG in pHERD30T | This study |
| PilB P228A PilA A86C + EV | mPAO1 PilB P228A PilA A86C with empty pHERD30T | This study |
| PilB P228A PilA A86C + Aqs1 YR:SG | mPAO1 PilB P228A PilA A86C with His_6_-Aqs1 YR:SG in pHERD30T | This study |
| mPAO1 + Tip | WT *P. aeruginosa* with His_6_-Tip in pHERD30T | This study |
| PilB P228A + Aqs1 | mPAO1 PilB P228A with His_6_-Aqs1 in pHERD30T | This study |
| PilB P228A + Tip | mPAO1 PilB P228A with His_6_-Tip in pHERD30T | This study |
| PilB P229A + Aqs1 | mPAO1 PilB P229A with His_6_-Aqs1 in pHERD30T | This study |
| PilB P229A + Tip | mPAO1 PilB P229A with His_6_-Tip in pHERD30T | This study |
| mNGr-PilZ + EV | mPAO1 with PilZ chromosomally tagged at the N-terminus with mNeonGreen with empty pHERD30T | This study |
| mNGr-PilZ + Aqs1 | mPAO1 with PilZ chromosomally tagged at the N-terminus with mNeonGreen and His_6_-Aqs1 in pHERD30T | This study |
| mNGr-PilZ + Aqs1 YR:SG | mPAO1 with PilZ chromosomally tagged at the N-terminus with mNeonGreen and His_6_-Aqs1 YR:SG in pHERD30T | This study |
| mNGr-PilZ + Tip | mPAO1 with PilZ chromosomally tagged at the N-terminus with mNeonGreen and His_6_-Tip in pHERD30T | This study |
| *pilB*::Tn5 + PilB N mut | mPAO1 *pilB* T5 transposon mutant with PilB L154N, L159N, L162N, and I164N in pHERD30T | This study |
| *pilB*::Tn5 + PilB^A152P G153P^ | mPAO1 *pilB* T5 transposon mutant with PilB A152P and G153P in pHERD30T | This study |
| *pilB*::Tn5 + PilB^D156P V157P^ | mPAO1 *pilB* T5 transposon mutant with PilB D156P and V157P in pHERD30T | This study |
| *pilB*::Tn5 + His_6_-PilB | mPAO1 *pilB* T5 transposon mutant with His_6_-PilB in pHERD30T | This study |
| *pilB*::Tn5 + His_6_-PilB D mut | mPAO1 *pilB* T5 transposon mutant with His_6_-PilB L154D, L159D, L162D in pHERD30T | This study |
| *pilB*::Tn5 + His_6_-PilB L232R | mPAO1 *pilB* T5 transposon mutant with His_6_-PilB L232R in pHERD30T | This study |
| *pilB*::Tn5 + His_6_-PilB I236R | mPAO1 *pilB* T5 transposon mutant with His_6_-PilB I236R in pHERD30T | This study |
| *pilB*::Tn5 + His_6_-PilB D mut L232R | mPAO1 *pilB* T5 transposon mutant with His_6_-PilB L232R L154D, L159D, L162D in pHERD30T | This study |
| *pilB*::Tn5 + His_6_-PilB D mut I236R | mPAO1 *pilB* transposon mutant with His_6_-PilB I236R L154D, L159D, L162D in pHERD30T | This study |
| mPAO1 + Aqs1 YR:SG W45A | mPAO1 with His_6_-Aqs1 YR:SG and W45A in pHERD30T | This study |
| Δ*gspE* + EV | mPAO1 Δ*gspE* + empty pHERD30T | This study |
| mPAO1 P229A + Aqs1 F19A | mPAO1 with chromosomal P229A mutation with His_6_-Aqs1 F19A in pHERD30T | This study |
| mPAO1 + pB | mPAO1 with empty pBADGr | This study |
| mPAO1+ pB-Aqs1 YR:SG | mPAO1 with Aqs1 YR:SG in pBADGr | This study |
| mPAO1 + pB-Aqs1 F19A | mPAO1 with Aqs1 F19A in pBADGr | This study |
| ***S. maltophilia* strains** | | |
| WCC C0169 + pB | *S. maltophilia* strain C0169 from the Wright clinical collection with empty pBADGr | This study |
| WCC C0169 + pB-Aqs1 YR:SG | *S. maltophilia* strain C0169 from the Wright clinical collection with Aqs1 YR:SG in pBADGr | This study |
| WCC C0169 + pB-Aqs1 F19A | *S. maltophilia* strain C0169 from the Wright clinical collection with Aqs1 F19A in pBADGr | This study |
| ***A. baylyi* strains** | | |
| ADP1 Parent | ADP1 *comP^T129C^* | ^5^ |
| P*_JExD_-aqs1* | ADP1 *comP*^T129C^ *∆vanAB::kan^R^,* P*_JExD_-his-aqs1* | This study |
| *∆pilB* | ADP1 *comP*^T129C^ *∆pilB* | ^5^ |
| *∆tfpB* | ADP1 *comP*^T129C^ *∆tfpB::gent^R^* | This study |
| *∆pilB ∆tfpB* | ADP1 *comP*^T129C^ *∆pilB ∆tfpB::kan^R^* | ^5^ |
| P*_JExD_-aqs1 ∆pilB* | ADP1 *comP*^T129C^ *∆pilB ∆vanAB::kan^R^,* P*_JExD_-his-aqs1* | This study |
| P*_JExD_-aqs1 ∆tfpB* | ADP1 *comP*^T129C^ *∆tfpB::gent^R^ ∆vanAB::kan^R^,* P*_JExD_-his-aqs1* | This study |
| **Plasmid list** | | |
| **Plasmid name** | **Characteristics** | **Source** |
| pEX18Gm | Suicide vector for gene replacement | ^6^ |
| pEX18Gm-Δ*lasR* | *lasR* deletion construct | This study |
| pEX18Gm-Δ*gspE* | *gspE* deletion construct | This study |
| pEX18Gm-PilB F187A | PilB F187A chromosomal knock-in construct | This study |
| pEX18Gm-PilB P228A | PilB P228A chromosomal knock-in construct | This study |
| pEX18Gm-PilB P229A | PilB P229A chromosomal knock-in construct | This study |
| pEX18Gm-PilA A86C | PilA A86C chromosomal knock-in construct | This study |
| pBADGr | Broad host range arabinose inducible expression vector | ^7^ |
| pBADGr-Aqs1 YR:SG | Aqs1 Y39S R40G in pBADGr | This study |
| pBADGr-Aqs1 F19A | Aqs1 F19A in pBADGr | This study |
| pHERD30T | Broad host range arabinose inducible expression vector | ^8^ |
| pHERD30T-Aqs1 | N-terminally His_6_-tagged Aqs1 in pHERD30T | ^2^ |
| pHERD30T-Tip | N-terminally His_6_-tagged Tip in pHERD30T | ^2^ |
| pHERD30T-Aqs1 YR:SG | N-terminally His_6_-tagged Aqs1 Y39S R40G in pHERD30T | This study |
| pHERD30T-Aqs1 F19A | N-terminally His_6_-tagged Aqs1 F19A in pHERD30T | This study |
| pHERD30T-Aqs1 W45A | N-terminally His_6_-tagged Aqs1 W4A in pHERD30T | This study |
| pHERD30T-Aqs1 YR:SG W45A | N-terminally His_6_-tagged Aqs1 Y39S, R40G, and W45A in pHERD30T | This study |
| PilB-T18 | mPAO1 full length PilB in pUT18C vector | This study |
| PilB-T25 | mPAO1 full length PilB in pKT25 vector | This study |
| N1D-T18 | mPAO1 PilB N1D in pUT18C vector | This study |
| N1D-T25 | mPAO1 PilB N1D in pKT25 vector | This study |
| N2D/CTD-T18 | mPAO1 PilB N2D and CTD in pUT18C vector | This study |
| N2D/CTD-T25 | mPAO1 PilB N2D and CTD in pKT25 vector | This study |
| LasR-T18 | mPAO1 LasR in pUT18C vector | This study |
| LasR-T25 | mPAO1 LasR in pKT25 vector | This study |
| Aqs1-T18 | mPAO1 Aqs1 in pUT18C vector | ^2^ |
| Aqs1-T25 | mPAO1 Aqs1 in pKT25 vector | ^2^ |
| Tip-T18 | mPAO1 Tip in pUT18C vector | ^2^ |
| Tip-T25 | mPAO1 Tip in pKT25 vector | ^2^ |
| PilB^Gm^-T18 | *G. metallireducens* PilB in pUT18C vector | This study |
| PilB^Gm^-T25 | *G. metallireducens* PilB in pKT25 vector | This study |
| PilB^Ab^-T18 | *A. baylyi* PilB in pUT18C vector | Howell Lab |
| PilB^Ab^-T25 | *A. baylyi* PilB in pKT25 vector | Howell Lab |
| TfpB-T18 | *A. baylyi* TfpB in pUT18C vector | Howell Lab |
| TfpB-T25 | *A. baylyi* TfpB in pKT25 vector | Howell Lab |
| MshE-T18 | *V. cholerae* MshE in pUT18C vector | This study |
| MshE-T25 | *V. cholerae* MshE in pKT25 vector | This study |
| CpaF-T18 | *C. crescentus* CpaF in pUT18 vector | Howell Lab |
| CpaF-T25 | *C. crescentus* CpaF in pKNT25 vector | Howell Lab |
| pHERD30T-PilB | Full length wildtype PilB in pHERD30T | ^4^ |
| pHERD30T-PilB F187A | Full length wildtype PilB F187A in pHERD30T | This study |
| pHERD30T-PilB P228A | Full length wildtype PilB P228A in pHERD30T | This study |
| pHERD30T-PilB P229A | Full length wildtype PilB P229A in pHERD30T | This study |
| pHERD30T-PilB L232A | Full length wildtype PilB L232A in pHERD30T | This study |
| pHERD30T-PilB I236A | Full length wildtype PilB I236A in pHERD30T | This study |
| pETDuet-ΔN1D (ΔD180)-PilB | N-terminally His_6_-tagged ΔD180-PilB first site and empty second site pETDuet-1 | This study |
| pETDuet-Aqs1 | Empty first site and N-terminally V5-tagged Aqs1 second site pETDuet-1 | This study |
| pETDuet-ΔN1D (ΔD180)-PilB/Aqs1 | N-terminally His_6_-tagged ΔD180-PilB first site and N-terminally V5-tagged Aqs1 second site pETDuet-1 | This study |
| pETDuet-ΔN1D-PilB F187A/Aqs1 | N-terminally His_6_-tagged ΔD180-PilB F187A first site and N-terminally V5-tagged Aqs1 second site pETDuet-1 | This study |
| pETDuet-ΔN1D-PilB P228A/Aqs1 | N-terminally His_6_-tagged ΔD180-PilB P228A first site and N-terminally V5-tagged Aqs1 second site pETDuet-1 | This study |
| pETDuet-ΔN1D-PilB P229A/Aqs1 | N-terminally His_6_-tagged ΔD180-PilB P229A first site and N-terminally V5-tagged Aqs1 second site pETDuet-1 | This study |
| pETDuet-ΔT148-PilB | N-terminally His_6_-tagged ΔT148-PilB first site and empty second site pETDuet-1 | This study |
| pETDuet- ΔT148-PilB/Aqs1 | N-terminally His_6_-tagged ΔT148-PilB first site and N-terminally V5-tagged Aqs1 second site pETDuet-1 | This study |
| pETDuet-PilB^Gm^ | N-terminally His_6_-tagged ΔN1D-*G. metallireducens* PilB first site and empty second site pETDuet-1 | This study |
| pETDuet-PilB^Gm^/Aqs1 | N-terminally His_6_-tagged ΔN1D-*G. metallireducens* PilB first site and N-terminally V5-tagged Aqs1 second site pETDuet-1 | This study |
| pETDuet-PilB^Ab^ | N-terminally His_6_-tagged ΔN1D-*A. baylyi* PilB first site and empty second site pETDuet-1 | This study |
| pETDuet-PilB^Ab^/Aqs1 | N-terminally His_6_-tagged ΔN1D-*A. baylyi* PilB first site and N-terminally V5-tagged Aqs1 second site pETDuet-1 | This study |
| PilB/PilZ pETDuet | N-terminally His_6_-tagged PilZ first site and full length PilB second site pETDuet-1 | This study |
| p15TVL-Aqs1 | N-terminally His_6_-tagged Aqs1 in p15TVL | ^2^ |
| p15TVL-Aqs1 W45A | N-terminally His_6_-tagged Aqs1 W45A in p15TVL | This study |
| pCDF-PilB | Full length un-tagged PilB in pCDF | This study |
| pETDuet-Aqs1/PilB | N-terminally His_6_-tagged Aqs1 first site and un-tagged full length PilB second site pETDuet-1 | This study |
| pACYDuet-PilZ | N-terminally His_6_-tagged PilZ first site and empty second site pACYDuet | This study |
| pHERD30T-PilB N mut | PilB L154N, L159N, L162N, and I164N in pHERD30T | This study |
| pHERD30T-PilB ^A152P G153P^ | PilB A152P and G153P in pHERD30T | This study |
| pHERD30T-PilB ^D156P V157P^ | PilB D156P and V157P in pHERD30T | This study |
| pHERD30T-His_6_-PilB | N-terminally His_6_-tagged PilB in pHERD30T | This study |
| pHERD30T-PilB D mut | N-terminally His_6_-PilB L154D, L159D, L162D in pHERD30T | This study |
| pHERD30T His_6_-PilB L232R | N-terminally His_6_-tagged PilB L232R in pHERD30T | This study |
| pHERD30T His_6_-PilB I236R | N-terminally His_6_-tagged PilB I236R in pHERD30T | This study |
| pHERD30T His_6_-PilB D mut L232R | N-terminally His_6_-tagged PilB L154D, L159D, L162D, and L232R in pHERD30T | This study |
| pHERD30T His_6_-PilB D mut I236R | N-terminally His_6_-tagged PilB L154D, L159D, L162D, and I236R in pHERD30T | This study |
