# Supplementary material for: A broad-spectrum phage-encoded mechanism to disarm bacterial type IV filaments
Source: PLoS Pathog. 2026 Jul 27;22(7):e1014478. doi: 10.1371/journal.ppat.1014478 (PMC13436744; doi:10.1371/journal.ppat.1014478)
Supplement: Supplementary file 3 [file ppat.1014478.s002.docx]

**S2 Table: Primers used in this study.**

| **Primers** | | |
| --- | --- | --- |
| **Primer name** | **Characteristics** | **Sequence** |
| PaPilB-T18/25_Fwd | PAO1 PilB for cloning into pUT18C and pKT25 | ATATTCTAGAGATGAACGACAGCATCCAACTG |
| PaPilB-T18/25_Rev | PAO1 PilB for cloning into pUT18C and pKT25 | ATATGGTACCTTAATCCTTGGTCACGCGG |
| GmPilB-T18/25_Fwd | *G. metallireducens* PilB for cloning into pUT18C and pKT25 | ATATGGATCCCATGCAGGCCAGCAGACTG |
| GmPilB-T18/25_Rev | *G. metallireducens* PilB for cloning into pUT18C and pKT25 | ATATGGTACCTTAATCGTCGGCCACGGTG |
| Pa PilB-N1D T18/25 | Encodes Pa PilB N1D with ΔD181-C terminus truncation for cloning into pUT18C and pKT25 | ATATGGTACCTTAGTCCGCTTCTGCGCTG |
| Pa PilB-N2D/CTD T18/25 | Encodes Pa PilB N2D and CTD with ΔD180-N terminus truncation for cloning into pUT18C and pKT25 | ATATTCTAGAGGACGCACCTGTAGTACGTTTCG |
| Pa PilB P183A_F | For making PilB P183A mutation | GCGGACGACGCAGCTGTAGTACGTTTCGTC |
| Pa PilB P183A_R | For making PilB P183A mutation | GACGAAACGTACTACAGCTGCGTCGTCCGC |
| Pa PilB F187A_F | For making PilB F187A mutation | CCTGTAGTACGTGCCGTCAACAAGATGCTG |
| Pa PilB F187A_R | For making PilB F187A mutation | CAGCATCTTGTTGACGGCACGTACTACAGG |
| Pa PilB P183A_F | For making PilB P228A mutation | CTCCACGAAGTGGCCAAGGCGCCGATCCAGTTGGCC |
| Pa PilB P183A_R | For making PilB P228A mutation | GGCCAACTGGATCGGCGCCTTGGCCACTTCGTGGAG |
| Pa PilB F187A_F | For making PilB P229A mutation | CACGAAGTGGCCAAGCCGGCGATCCAGTTGGCC |
| Pa PilB F187A_R | For making PilB P229A mutation | GGCCAACTGGATCGCCGGCTTGGCCACTTCGTG |
| Pa PilB L232A_F | For making PilB L232A mutation | GTCGCCAAGCCGCCGATCCAGGCCGCCAGTCGTATCTCTGCTCGTC |
| Pa PilB L232A_R | For making PilB L232A mutation | GACGAGCAGAGATACGACTGGCGGCCTGGATCGGCGGCTTGGCGAC |
| Pa PilB I236A_F | For making PilB L236A mutation | GTCGCCAAGCCGCCGATCCAGTTGGCCAGTCGTGCCTCTGCTCGTCTCAAGGTA |
| Pa PilB I236A_R | For making PilB L236A mutation | TACCTTGAGACGAGCAGAGGCACGACTGGCCAACTGGATCGGCGGCTTGGCGAC |
| PilBPa F187A chrom mut_F | For knocking F187A onto the chromosome | GCACCTGTAGTACGCGCCGTCAACAAGATGCTGCT |
| PilBPa F187A chrom mut_R | For knocking F187A onto the chromosome | AGCAGCATCTTGTTGACGGCGCGTACTACAGGTGC |
| PilB F187A Ups Fwd | For amplifying region upstream of PilB F187 | ATATTCTAGACTACTCGACGAAAAGACCGCC |
| PilB F187A Dns Rev | For amplifying region downstream of PilB F187 | ATATGGTACCGTTCAGGCCGGTGTATAGCG |
| PilBPa P228A chrom mut_F | For knocking P228A onto the chromosome | GTCGCCAAGGCGCCGATCCAGTTGGCCAGT |
| PilBPa P228A chrom mut_R | For knocking P228A onto the chromosome | ACTGGCCAACTGGATCGGCGCCTTGGCGAC |
| PilB P228A Ups Fwd | For amplifying region upstream of PilB P228 | ATATTCTAGAGAGCAGTTCGGCATCGCCTATT |
| PilB P228A Dns Rev | For amplifying region downstream of PilB P228 | ATATGGTACCCGCCGACCATGATCACATCC |
| PilBPa P229A chrom mut_F | For knocking P229A onto the chromosome | CACGAAGTCGCCAAGCCGGCGATCCAGTTGGCCAGTC |
| PilBPa P229A chrom mut_R | For knocking P229A onto the chromosome | GACTGGCCAACTGGATCGCCGGCTTGGCGACTTCGTG |
| PilB P229A Ups Fwd | For amplifying region upstream of PilB P229 | ATATTCTAGACCGAGCAGTTCGGCATCG |
| PilB P229A Dns Rev | For amplifying region downstream of PilB P229 | ATATGGTACCTCTCGCCGACCATGATCACA |
| PilB N2D/CTD_Fwd | Encodes Pa PilB N2D and CTD with ΔD180-N terminus truncation for cloning into pET28b | ATATGCTAGCGACGCACCTGTAGTACGTTTCGTC |
| Pa PilB_Rev | For cloning PilB into pET28b | ATATGAATTCTTAATCCTTGGTCACGCGGTT |
| Aqs1 pBAD_Fwd | For cloning Aqs1 into pBADGr | ATATGAATTCATGACAAACACCGACCTCAAAC |
| Aqs1 pBAD_Rev | For cloning Aqs1 into pBADGr | ATATAAGCTTTCATTCATGGTCGCCCC |
| LasR-T18/25_Fwd | For cloning full length PAO1 LasR into pUT18C and pKT25 | ATATGGATCCCATGGCCTTGGTTGACG |
| LasR-T18/25_Rev | For cloning full length PAO1 LasR into pUT18C and pKT25 | ATATGGTACCTCAGAGAGTAATAAGACCCAAATTAAC |
| PilB N2DDuet_F | Encodes Pa PilB N2D and CTD with ΔD180-N terminus truncation for cloning into pETDuet-1 first site | ATATGAATTCGGACGCACCTGTAGTACGTTTCGTC |
| PilB N2DDuet_R | Encodes Pa PilB N2D and CTD with ΔD180-N terminus truncation for cloning into pETDuet-1 first site | ATATGCGGCCGCTTAATCCTTGGTCACGCGGTT |
| Aqs1 V5 Tag pETDuet_F | For cloning Aqs1 with a V5 tag into pETDuet-1 | ATATCAATTGGGGCAAACCGATTCCGAACCCGCTGCTGGGCCTGGATAGCACCATGACAAACACCGACCTCAAAC |
| Aqs1 V5 Tag pETDuet_R | For cloning Aqs1 with a V5 tag into pETDuet-1 | ATATGGTACCTCATTCATGGTCGCCCC |
| PilB pHERD_Fwd | For cloning PilB into pHERD30T | ATATGAGCTCGCGATTCCTTCCCCATGA |
| PilB pHERD_Rev | For cloning wild-type PilB into pHERD30T | ATATTCTAGATTAATCCTTGGTCACGCGG |
| PilA A86C Fwd | For mutating PAO1 PilA A86C | GGCGTCGAGCCGGATTGTAACAAGTTGGGTGTA |
| PilA A86C Rev | For mutating PAO1 PilA A86C | TACACCCAACTTGTTACAATCCGGCTCGACGCC |
| PilA A86 Ups_Fwd | PilA A86 primer for upstream region amplification | ATATGAATTCCGCTCAGTTGGATGCTGTC |
| PilA A86 Dns_Rev | PilA A86 primer for downstream region amplification | ATATTCTAGAGCCAAGCTGGAAGCTTCC |
| LasR UpS_F | For making a *lasR* deletion upstream region | ATATGAATTCGGGGACCAGGTGTGACTG |
| LasR UpS_R | For making a *lasR* deletion upstream region | ATATGGATCCAGGATGGCGCTCCACTC |
| LasR DnS_F | For making a *lasR* deletion downstream region | ATATGGATCCCGGAAGTTCGGTGTGACC |
| LasR DnS_R | For making a *lasR* deletion downstream region | ATATAAGCTTCCATCGATTTCCATCTCGTC |
| GspE Ups_F | For making a *gspE* deletion upstream region | ATATGAATTCCTGCGGATCGACTCGTGG |
| GspE Ups_R | For making a *gspE* deletion upstream region | ATATTCTAGAGCGAAGCTGAACGGCAGAC |
| GspE Dns_F | For making a *gspE* deletion downstream region | ATATTCTAGACAAGGTGCTGGAAGGCGTC |
| GspE Dns_R | For making a *gspE* deletion downstream region | ATATAAGCTTTGCTCGGTGTAGTCGGCAAG |
| mNGr-PilZ_F1 | For cloning a PilZ mNeonGreen N-terminal fusion | ATATGAATTCGGCAGGAACCTGCATGGTGAGCAAGGGCGAGGAG |
| mNGr-PilZ_R1 | For making an mNeonGreen-PilZ N-terminal fusion construct - encodes 5G linker | ACCACCACCACCACCCTTGTACAGCTCGTCCATGCC |
| mNGr-PilZ_F2 | For cloning a PilZ mNeonGreen N-terminal fusion | GGTGGTGGTGGTGGTATGAGTTTGCCACCCAATCTG |
| mNGr-PilZ_R2 | For cloning a PilZ mNeonGreen N-terminal fusion | ATATAAGCTTTTACATCGTGTGGGTCGGC |
|  |  |  |
| PilB F187ADuet_F | For cloning PilB F187A into pETDuet-1 | ATATGAATTCGGACGCACCTGTAGTACGTGCCGTC |
| PilB linker N mut_F | Mutates all L and I residues between A147-179 to N | AACGCAGGCAACGACGATGTCGACAACGAGGGAAATGATAAC |
| PilB linker N mut_R | Mutates all L and I residues between A147-179 to N | GTTATCATTTCCCTCGTTGTCGACATCGTCGTTGCCTGCGTT |
| PilB ^A152P G153P^ linker_F | Mutates PilB A152 and G153 to P | ACCGACGGTCTCCCACCCCTCGACGATGTCGAC |
| PilB ^A152P G153P^ linker_R | Mutates PilB A152 and G153 to P | GTCGACATCGTCGAGGGGTGGGAGACCGTCGGT |
| PilB ^D156P V157P^ linker_F | Mutates PilB D156 and V157 to P | CGCAGGCCTCGACCCTCCCGACCTCGAGGGACTGG |
| PilB ^D156P V157P^ linker_R | Mutates PilB D156 and V157 to P | CCAGTCCCTCGAGGTCGGGAGGGTCGAGGCCTGCG |
| MshE-T18/25_F | For cloning *mshe* into BACTH T18/T25 vectors | ATATTCTAGAGGTGCCAATTAATAAACTGCGTAAAC |
| MshE-T18/25_R | For cloning *mshe* into BACTH T18/T25 vectors | ATATGAATTCCTACAGATAAATCGGTTCAACCAA |
| N-His PilB 30T_F | For cloning PilB with an N-terminal His-tag into pHERD30T | ATATGAGCTCATTCCTTCCCCATGCATCATCATCATCATCACAACGACAGCATCCAA |
| PilB I236R_F | For making a PilB I236R mutation | ATCCAGTTGGCCAGTCGTCGCTCTGCTCGTCTCAAGG |
| PilB I236R_R | For making a PilB I236R mutation | CCTTGAGACGAGCAGAGCGACGACTGGCCAACTGGAT |
| PilB L232R_F | For making a PilB L232R mutation | GCCAAGCCGCCGATCCAGCGGGCCAGTCGTATCTCTG |
| PilB L232R_R | For making a PilB L232R mutation | CAGAGATACGACTGGCCCGCTGGATCGGCGGCTTGGC |
| PilB L154D_F | For making a PilB L154D mutation | CCGACGGTCTCGCAGGCGACGACGATGTCGACCTCG |
| PilB L154D_R | For making a PilB L154D mutation | CGAGGTCGACATCGTCGTCGCCTGCGAGACCGTCGG |
| PilB L159D_F | For making a PilB L159D mutation | CTCGACGATGTCGACGACGAGGGACTGGATATC |
| PilB L159D_R | For making a PilB L159D mutation | GATATCCAGTCCCTCGTCGTCGACATCGTCGAG |
| PilB L162D_F | For making a PilB L162D mutation | GTCGACCTCGAGGGAGACGATATCGGTAGCGCGGAC |
| PilB L162D_R | For making a PilB L162D mutation | GTCCGCGCTACCGATATCGTCTCCCTCGAGGTCGAC |
| PilB Linker D mut_F | For making a PilB L154D, L159D, and L162D mutant | CTCGCAGGCGACGACGATGTCGACGACGAGGGAGACGATATCGGTAGCGCGGAC |
| PilB Linker D mut_F | For making a PIlB L154D, L159D, and L162D mutant | GTCCGCGCTACCGATATCGTCTCCCTCGTCGTCGACATCGTCGTCGCCTGCGAG |
| PilB linker T18/25_F | For cloning PilB linker T148-D180 into T18 and T25 | ATATTCTAGAGACCGACGGTCTCGCAGGC |
| PilB linker T18/25_R | For cloning PilB linker T148-D180 into T18 and T25 | ATATGAATTCTTAGTCCGCTTCTGCGCTGG |
| PilB Ab N2D/CTD Duet_F | For cloning *A. baylyi* PilB from K188 into pETDuet-1 | ATATGAATTCGAAACTTAAAGACGAAGCACCTATTG |
| PilB Ab N2D/CTD Duet_R | For cloning *A. baylyi* PilB from K188 into pETDuet-1 | ATATAAGCTTTTATTCACTGGTTACACGATTAATTTC |
| PilB Gm N2D/CTD Duet_F | For cloning *G. metallireducens* PilB from D180 into pETDuet-1 | ATATGAATTCGGACGCTCCGGTCGTGAAG |
| PilB Gm N2D/CTD Duet_R | For cloning *G. metallireducens* PilB from D180 into pETDuet-1 | ATATCTGCAGTTAATCGTCGGCCACGGT |
